# Acute Hemorrhagic Rectal Ulcer Syndrome: A Known Yet Often Overlooked Etiology of Gastrointestinal Bleeding in Chronically Ill Patients

**DOI:** 10.7759/cureus.26932

**Published:** 2022-07-17

**Authors:** Yasir Rajwana, Kosisochukwu J Ezeh, Etan Spira

**Affiliations:** 1 Internal Medicine, Jersey City Medical Center, Jersey City, USA; 2 Gastroenterology and Hepatology, Jersey City Medical Center, Jersey City, USA

**Keywords:** ano rectal diseases, cauterization, acute gastrointestinal bleed, acute hemorrhagic rectal ulcer syndrome, ahrus

## Abstract

Acute Hemorrhagic Rectal Ulcer Syndrome (AHRUS) is a known and potentially overlooked cause of severe gastrointestinal bleeding in patients with critical illness. It presents as a sudden and brisk painless bleed. It is common among elderly patients who have chronic conditions such as coronary artery diseases associated with the use of anti-platelets, diabetes mellitus, hypoalbuminemia, liver diseases, sepsis, stroke, and chronic renal failure on hemodialysis. AHRUS could result in fatal gastrointestinal hemorrhage. Here, we report a case of acute hemorrhagic rectal ulcer with the above-mentioned risk factors and make the argument that AHRUS should be an important differential in a similar population presenting with a gastrointestinal bleed.

## Introduction

Acute hemorrhagic rectal ulcer syndrome (AHRUS) is a rare entity that has been mostly described in the Eastern hemisphere for a long time. The term was introduced in 1981 in Japan [1], described in patients with acute onset of painless and massive rectal bleeding. It is usually diagnosed via careful examination with colonoscopy of the rectum and ano-rectum for mucosal ulceration. It is variable in characteristics; It may be solitary or multiple. It is characterized by sudden massive rectal bleeding in elderly patients with various comorbidities. There have been a few epidemiological studies investigating risk factors of AHRUS, which are discussed later.

As it remains under-recognized in the Western hemisphere, increased awareness is required to influence the outcome of massive lower GI bleeding in critically ill patients. This lack of awareness may contribute to delays in making the diagnosis and in instituting preventive measures. Given the characteristic of patients with this pathology, it should be considered a potential lethal cause of lower gastrointestinal (GI) bleed in hospitalized patients. 

This article was previously presented as a meeting abstract at the 2021 American College of Gastroenterology Annual Scientific Meeting on October 27, 2021.

## Case presentation

A 63-year-old male with a history of decompensated liver cirrhosis secondary to alcohol use, coronary artery disease, and hypertension presented with a day history of painless rectal bleed. He had no history of previous GI bleeding. He had been on dual anti-platelet therapy (aspirin and clopidogrel) for medical management of triple vessel disease deemed inoperable due to advanced liver disease. He had no prior colonoscopy nor upper endoscopy. He denied abdominal pain, nausea, vomiting, constipation, fever, and chills.

On presentation, although directable, he had altered mental status, Glasgow coma scale of 12, and his brother confirmed that he usually is taking rifaximin for encephalopathy and his mental status would wax and wane, which is his baseline mentation. Physical examination was remarkable for jaundice, positive orthostasis (as interpreted by a change in heart rate from lying to sitting position), and large blood clot protruding from the rectum. Initial vital signs were significant for elevated heart rate and decreased mean arterial pressure lower than 65. Initial laboratory values showed Hgb 12.5g/dl, mean corpuscular volume (MCV) 100.2, hematocrit (HCT) 38.4%, WBC 9.8 k/uL, blood glucose was 161 mg/dl, prothrombin time (PT) 14.4s, international normalized ratio (INR) 1.14, PTT 40.3s, albumin 3.5g/dl, aspartate aminotransferase (AST) 108 units/L, alanine transaminase (ALT) 58 Units/L, alkaline phosphatase (ALP) 629 units/L. Total bilirubin 4.6 mg/dl, direct bilirubin 3 mg/dl, blood urea nitrogen (BUN)/creatinine (Cr) 36/2.21, and sodium 123 mmol/L as depicted in Table 1. Given concerns for hemorrhagic shock secondary to GI bleed, he was admitted to the intensive care unit.

**Table 1:** Initial laboratory results BUN: blood urea nitrogen; Cr: creatinine; PT: prothrombin time; PTT: partial thromboplastin time; INR: international normalized ratio; AST: aspartate aminotransferase; ALT: alanine transaminase; ALP: alkaline phosphatase

Laboratory parameters	Values	Normal ranges
Hemoglobin/Hematocrit	12.5 g/dL / 38.4%	14-18 g/dL / 42-52%
Platelet	229 k/uL	130-400 k/uL
White blood cells	9.8 k/uL	4.5-11 k/uL
Serum sodium	123 mmol/L	136-145 mmol/L
BUN	36 mg/dL	9-23 mg/dL
Cr	2.21 mg/dL	0.7-1.3 mg/dL
PT	14.4s	11.8-13.9s
PTT	40.3s	22.9-37.7s
INR	1.14	0.89-1.09
Total Protein	6.3 g/dL	5.7-8.2 g/dL
Albumin	3.5 g/dL	3.2-4.8 g/dL
Total Bilirubin	4.6 mg/dL	0.2-1.1 mg/dL
Direct Bilirubin	3 mg/dL	0.1-0.3 mg/dL
AST	108 units/L	8-34 units/L
ALT	58 units/L	10-49 units/L
ALP	629 units/L	46-116 units/L
Serum glucose	161 mg/dL	74-106 mg/dL

**Figure 1:**
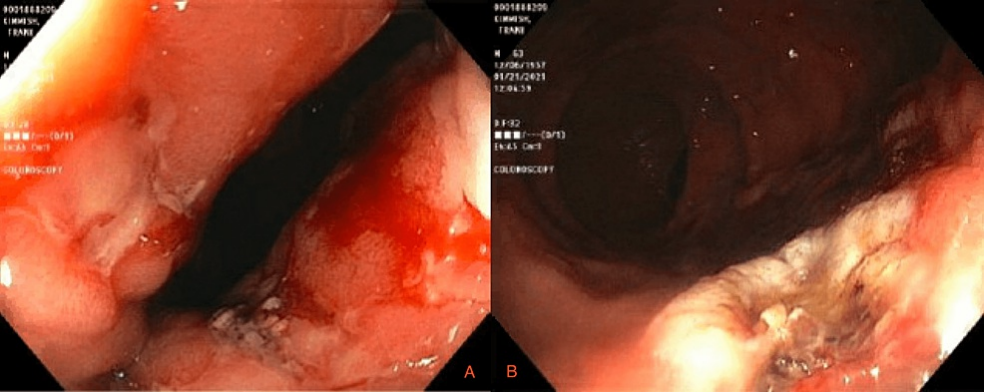
A. Rectal ulcer; B. Rectal ulcer following gold probe application

He was initially started on octreotide due to decompensated liver disease as it was unable to discern if the bleed was from a brisk upper GI bleed. Resuscitation measures were initiated and anti-platelet was held. He required multiple blood transfusions (~4pRBCs) due to a precipitous fall in hemoglobin (6.9) and hypotension. The gastroenterology team was on board, with an initial recommendation to stabilize the patient. The patient continued to have multiple bright red, large volume bloody bowel movements. He was wheeled to the endoscopy suite given active GI bleed. Upper endoscopy (EGD) revealed no varices, ulcers, arteriovenous malformations, portal GI, or active bleeding source. Colonoscopy showed a large amount of clotted blood that, when removed, revealed two rectal ulcers in the distal rectum at the anal verge with associated granular rectal mucosa as seen in Figure 1, with one showing stigmata of recent bleeding. Complete visualization of the colon revealed a small polyp of 7mm, which was excised with no other lesions or source of bleeding except for the ulcer noted above. It was subsequently cauterized with a gold probe resulting in the cessation of further bleeding. 

The patient required no further blood transfusions following the intervention. Repeat laboratory prior to discharge was hemoglobin 9.8 g/dL, serum sodium 133 mmol/L, improved kidney function BUN/Cr 26/1.35. He was subsequently discharged after monitoring for three days inpatient to follow up outpatient with his gastroenterology and hepatology as well as cardiology team. He was cleared to resume aspirin only seven days thereafter. 

## Discussion

**Figure 2:**
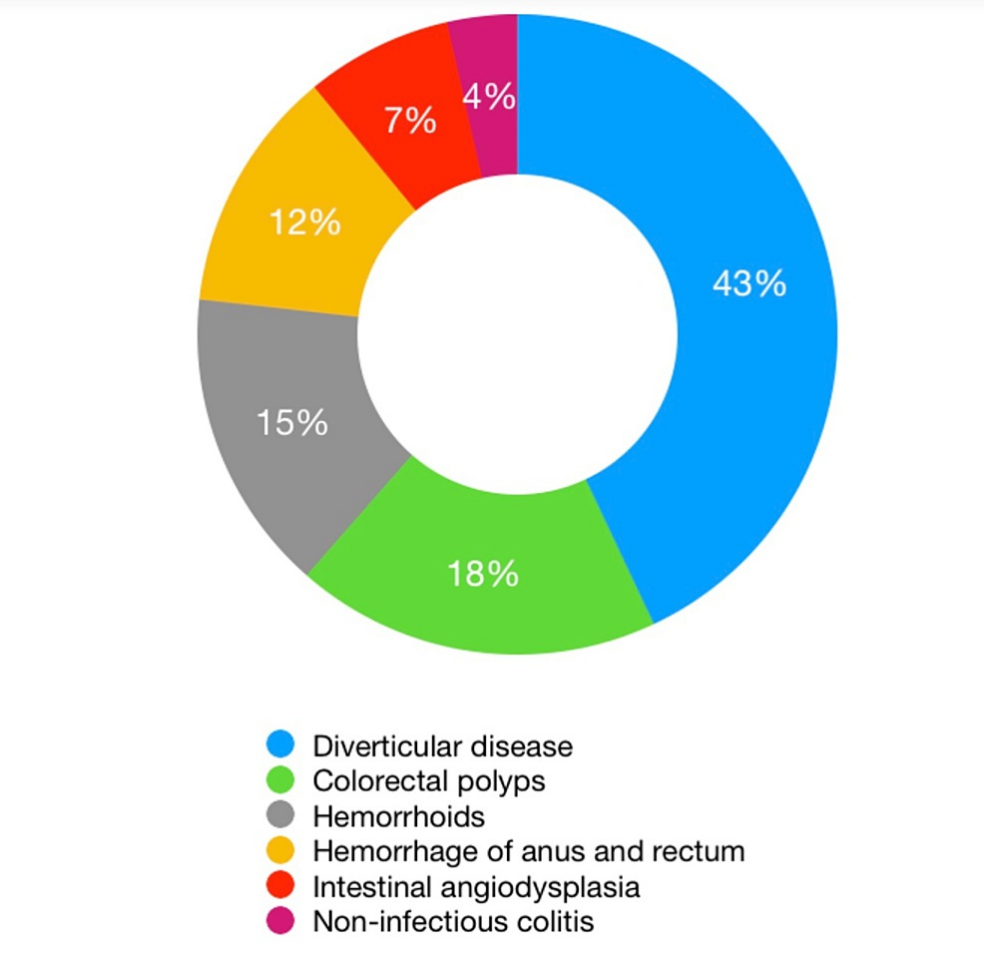
Epidemiology of lower gastrointestinal bleeds

GI bleed is a common gastrointestinal cause of hospitalization. It is divided into upper and lower GI bleed based on the location, i.e., proximal or distal to the ligament of Treitz. Lower GI bleed involves a wide range of clinical presentations from minor to major hemorrhage with shock. Incidence increases with age, accounting for about approximately 20 to 30 hospitalizations per 100,000 adults per year [1]. Figure 2 illustrates the epidemiology of lower GI bleeds, with AHRUS among the least common [2].

AHRUS was first noted in Japan [3]. The characteristics of patients described with this pathology have included older age, immobility, anti-thrombotic drug use, and comorbidities such as diabetes mellitus, coronary artery disease, cerebrovascular attacks, sepsis, liver failure, hypoalbuminemia, and chronic renal failure with hemodialysis [4]. It manifests as a painless massive lower GI bleed. 

Several diagnostic criteria have been developed by studies such as 1) abrupt massive painless rectal bleeding, 2) serious comorbidities as mentioned above, 3) colonoscopy finding of ulceration with stigmata of bleed, 4) no history of non-steroidal anti-inflammatory drug, and 5) absence of upper GI tract bleed with upper endoscopy [5]. All of these criteria were fulfilled by our patient. It is characterized histopathologically by necrosis with striping of epithelium with multiple thrombi in the vessel findings [5].

Sugawa et al. revealed that among the problems in diagnosing AHRUS is differentiating this disorder from other rectal ulcers hemorrhage as the endoscopic visualization of some stercoral lesions appears the same as those of AHRUS [6]. However, the clinical setup is considerably diverse, and caution is needed. The stercoral lesions relate to serious constipation and the stool is very hard at the edge of the lesion [5]. 

Additionally, ischemic lesions might also be hard to differentiate from AHRUS endoscopically. Montoro et al. (2011) revealed that the ischemic lesions have a high likelihood of being linked to vascular impact on other GI areas and present with typical computed tomography (CT) scan results of ischemic bowel [7]. Hence, proper history is needed to help distinguish AHRUS from other similar pathologies. Lin et al. go further to state that AHRUS ought to be regarded as a significant cause of acute hemorrhage in the lower GI in patients with critical illnesses [8]. These people usually have other related diseases such as diabetes mellitus, lung failure, renal failure, and others. Colonoscopy could aid in the early detection of the condition, treatment, and management. The management of people who have AHRUS is based on the right diagnosis and treatment of the underlying condition.

Current endoscopic hemostasis has proven to be adequate, although associated with rebleeding. In our case, the patient remained stable with no recurrence following cauterization with the gold probe. Other modalities that exist are trans-anal gauze tamponade [9] and trans-anal suture ligation [7].

## Conclusions

AHRUS occurs in elderly, critically ill, and bedridden patients with sudden, severe, painless, rectal bleeding and is managed by correction of coagulopathy and by endoscopic hemostasis. Early identification of these risk factors in the evaluation of lower GI bleed could make clinicians more aware of the possibility of AHRUS. It is an important differential diagnosis to consider in elderly populations, especially with the risk factors described, besides common causes like diverticulosis as early recognition can lead to timely therapeutic intervention. 
